# Bidirectional association between ESRD dialysis and diabetes: National cohort study

**DOI:** 10.1371/journal.pone.0173785

**Published:** 2017-03-15

**Authors:** Yeh-Wen Chu, Wen-Shiann Wu, Chen-Fang Hsu, Jhi-Joung Wang, Shih-Feng Weng, Chih-Chiang Chien

**Affiliations:** 1 Department of Nephrology, Chi-Mei Medical Center, Tainan, Taiwan; 2 Department of Cardiology, Chi-Mei Medical Center, Tainan, Taiwan; 3 Department of Pharmacy, Chia-Nan University of Pharmacy and Science, Tainan, Taiwan; 4 Departments of Pediatrics, Chi -Mei Medical Center, Tainan, Taiwan; 5 Department of Medical Research, Chi-Mei Medical Center, Tainan, Taiwan; 6 Department of Healthcare Administration and Medical Informatics, Kaohsiung Medical University, Kaohsiung, Taiwan; Universita degli Studi di Perugia, ITALY

## Abstract

**Background:**

Diabetes is associated with development of end-stage renal disease (ESRD) dialysis, but it is not clear whether ESRD dialysis is a risk factor for new-onset diabetes (NODM).

**Methods:**

Using the Taiwan National Health Insurance Research Database, we designed two cohort studies to determine the association between dialysis and diabetes. Analysis 1 estimated the hazard ratios (HR) of ESRD dialysis in 20,585 patients with type 2 diabetes (T2DM) and 82,340 gender- and age- matched controls without diabetes. Analysis 2 estimated the HRs of NODM in 18,489 ESRD patients undergoing dialysis and 73,956 gender- and age- matched controls without ESRD dialysis. The follow-up period was from 2000 to date of endpoint, the date of death, or December 31, 2008. Cox proportional models were used to estimate the relative hazards.

**Results:**

In analysis 1, the incidence of ESRD dialysis was higher in the T2DM cohort than in the non-diabetes cohort (6.78 vs. 0.61 per 1,000 person-years; HR: 7.97; 95%CI: 7.05–8.00). In analysis 2, the incidence of NODM was higher in the ESRD dialysis cohort than in the without-ESRD dialysis cohort (22.84 vs. 13.99 per 1,000 person-years; HR: 1.40; 95% CI: 1.34–1.47).

**Conclusions:**

ESRD dialysis and diabetes were bidirectionally associated. The relationship between T2DM and incident ESRD dialysis was much stronger than between ESRD dialysis and NODM. Further studies are needed to determine the mechanism of ESRD dialysis-related NODM.

## Introduction

End-stage renal disease (ESRD) is an important complication of diabetes (DM) [1]. Many studies report an association between pre-existing DM at the initiation of dialysis and a poor outcome in ESRD patients undergoing dialysis [1, 2]. In recent years, new-onset diabetes mellitus (NODM) after the initiation of dialysis has been reported in some studies [3–6].

The prevalence of DM is higher in ESRD dialysis patients than in the general population [1, 4, 5]. The associations may be related to an increased risk of ESRD dialysis in individuals with DM [1], an increased risk of DM in ESRD dialysis patients [4–6], or both. Several studies have reported that elevated NODM occurs among patients receiving dialysis at rates greater than in the general population [3, 5]. According to the U.S. Center for Disease Control [7], the incidence rate for DM in the general population in the U.S. fluctuated about 0.5–0.9% per year from 2000–2009. Using data from the U.S. Renal Data System in a three-year follow-up study, the incidence of NODM after dialysis was 20 per 1,000 patient-years and its prevalence to be 4.6% in the first year after initiation of dialysis [5]. In Taiwan, the incidence of DM in the general population from 2000 to 2009 was 0.7–0.9% per year [8]. A recent study [4] that used Taiwan`s national health insurance claims for ESRD dialysis patients found the cumulative incidence rate of NODM to be 4% at one year, 9% at three years, and 14% at five years. Although the underline mechanism for the relationship between ESRD dialysis and NODM is not fully understood, the factors associated with ESRD dialysis, including being older, having a cardiovacualr cardiovascular disease, a stroke, heart failure, et al [4], and being in a state of chronic inflammation [6], are associated with insulin resistance and the development of type 2 diabetes (T2DM).

Many studies have evidenced DM as a strong predictor of ESRD dialysis [2]; however, there are no published studies on the bidirectional association between DM and ESRD dialysis. The worldwide numbers of patients with ESRD and patients with DM have both grown significantly in recent decades [9]. Knowledge of the strength of that bidirectional relationship between DM and ESRD dialysis might help us develop preventive strategies intended to reduced comorbid diabetes in ESRD dialysis patients. Taiwan has a very high prevalence and incidence of ESRD dialysis [10]. In this article, we used a large data set from the Taiwan National Health Insurance Research Database (NHIRD) to test whether patients with baseline T2DM predicted incident ESRD dialysis and whether patients with ESRD dialysis at baseline were more likely to develop NODM over follow-up than were patients without it.

## Materials and methods

### Database

The National Health Insurance (NHI) program has provided compulsory universal health insurance in Taiwan since 1995. With the exception of prison inmates, all citizens are enrolled in the program. All contracted medical institutions must submit standard computerized claim documents for medical expenses. Patients with ESRD are eligible for any type of renal replacement therapy free of any charge. All patients undergoing chronic dialysis are covered by NHI.

The data analyzed in this study were obtained from two databases in the National Health Insurance Research Database (NHIRD) and released for research by the Taiwan National Health Research Institute. T2DM cohort included as the subjects were identified from the representative database (NHRI-NHIRD-100057); the database contains claims of 1 million beneficiaries randomly selected from all beneficiaries insured in the year 2000, with age and gender distributions nearly identical to the entire insured population of Taiwan, which represents approximately 5% of Taiwan`s population. ESRD dialysis cohort included as the subjects were identified from the representative database (NHRI-NHIRD-99182); the database contains medical claims and information of all ESRD patients on maintenance dialysis in Taiwan. ESRD patients on maintenance dialysis were defined as having undergone dialysis for more than 90 days.

The NHIRD covers nearly all (99%) inpatient and outpatient medical benefit claims for Taiwan’s 23 million residents, is one of the largest and most comprehensive databases in the world, and has been used extensively in various studies [1, 4, 11, 12]. Patient gender, birthday, ambulatory care visit claims, dates of admission and discharge, medical institutions providing the services, the ICD-9-CM (International Classification of Diseases, 9th Revision, Clinical Modification) diagnostic and procedure codes (up to five each), and outcomes are encrypted. Urbanization level was based on an NHRI study and modified from 7 levels into the following 3 categories: urban for levels 1 to 2, suburban for levels 3 to 4, and rural for levels 5 to 7 [13].

### Cohort analysis of the association between type 2 diabetes and risk of incident ESRD dialysis

#### T2DM cohort

Adult patients (≥ 18 years old) with T2DM (ICD-9-CM: 250X0 or 250X2) were identified according to one of the definitions below: (1) Diagnostic codes at any time in outpatient visits of the year 2000 in the representative database (NHRI-NHIRD- 100057) and then experienced another one or more diagnoses within the subsequent 12-month follow-up period. The first and last outpatient visit within 1 year had to be more than 30 days apart to avoid accidental inclusion of miscoded patients; (2) Diagnostic codes had to be in the representative database (NHRI-NHIRD- 100057) during hospitalization at least once in the year 2000 [4]. The patients were excluded if they had a history of ESRD on chronic dialysis or renal transplantation before their first visit for diabetes in 2000. In all, 20,585 patients with diabetes but not on ESRD dialysis were included in the T2DM group.

#### Non-diabetes (control) cohort

An age- and gender- matched (with the T2DM cohort) group without DM was randomly selected from all beneficiaries who were free from both diabetes and ESRD dialysis in 1998–2000. Case matching was done with the gmatch SAS macro (Developed by Erik Bergstralh and Jon Kosanke, 2003, Mayo Clinic). It computerized matching of cases to controls using the greedy matching algorithm with a fixed number of controls per case. The controls may be matched to the cases by one or more factors (age and gender in our study). The control selected for a particular case(i) will be the control(j) closest to the case in terms of Dij. Dij can be defined in multiple ways. Common choices are the Euclidean distance and the weighted sum of the absolute differences between the case and control matching factors. In our study, the Controls-to-Cases matching ratio was 4:1 (n = 82,340).

Patients were followed-up from the index date in 2000 to the initiation of incident ESRD dialysis, the date of death, or December 31, 2008. We evaluated the incidence of ESRD dialysis. The index data for each patient in the diabetes group was the date of his or her first diabetes diagnosis. The index date for patients in the control group was the first date of enrollment in the NHI program. If the first date of enrollment was before January 2000, the index date was set as 1 January 2000.

The age- and gender-specific hazard rates were determined with person-years as the denominator under the Poisson assumption. The cumulative incidence of ESRD dialysis was calculated. Cox proportional hazards regression models were used to assess the association of T2DM with the risk of incident ESRD dialysis. The independent associations were examined using multivariate analysis with adjusting simultaneously for age, gender, geographic area, urban/rural status, and various comorbidities. We adjusted geographic variables for the presence of an urban-rural difference in the accessibility to medical care in Taiwan [13]. We linked to the diagnostic codes through the inpatient and outpatient claims databases of the NHI. Baseline comorbidities, including hypertension (HTN), congestive heart failure (CHF), coronary artery disease (CAD), cerebrovascular accident (CVA), hyperuricemia and dyslipidemia, are important risk factors for ESRD dialysis and were assessed at baseline. These characteristics were consistent with those in previous studies [14–20] and demonstrate the need to adjust when comparing rates of ESRD dialysis. The ICD-9-CM codes used to define each comorbidity: 1) HTN (362.11, 401.x -405.x, 437.2); 2) CHF (428, 402.x1, 404.x1, 404.x3, 398.91); 3) CAD (410.xx - 414.xx); 4) CVA (430-438.xx); 5) hyperuricemia (274.xx); 6) dyslipidemia (272.0, 272.2, 272.4). Those comorbidities were identified according to one of the definitions below: (1) Diagnostic codes at any time in outpatient visits of the year 2000 in the representative database and then experienced another one or more diagnoses within the subsequent 12-month follow-up period. The first and last outpatient visit within 1 year had to be more than 30 days apart to avoid accidental inclusion of miscoded patients; (2) Diagnostic codes had to be in the representative database during hospitalization at least once in the year 2000. The method of identifying these comorbidities have been used extensively in various studies of Taiwan National Health Research Institute and many articles have been published [1, 4, 11, 12]. All statistical analyses were done using the Statistical Package for Social Sciences 17.0 for Windows (SPSS Inc; Chicago, IL, USA). Significance was set at p < 0.05.

### Cohort analysis of the association between ESRD dialysis and risk of new-onset diabetes

#### ESRD dialysis cohort

For this longitudinal cohort study, we selected only adult patients (≥ 18 years old) in the ESRD database (NHRI-NHIRD-99182) and on maintenance dialysis between January 1 and December 31, 2000. The patients were excluded if they had a history of DM or renal transplantation before their first visit for dialysis in 2000. In all, 18,489 patients on ESRD dialysis without DM were included in the ESRD dialysis group.

#### Non-dialysis (control) cohort

An age- and gender- matched (with the ESRD dialysis cohort) group not on ESRD dialysis was randomly selected from all beneficiaries in the representative database (NHRI-NHIRD-100057) who were free from both ESRD dialysis and DM in 1998–2000. The Control-to-Case matching ratio was 4:1 (n = 73,956) to maximize power.

Patients were followed-up from the index date in 2000 to NODM, the date of death, the end of dialysis, or December 31, 2008. We evaluated the incidence of NODM. We linked to the diagnostic codes through the inpatient and outpatient claims databases. Cases of type 2 diabetes (ICD-9-CM: 250X0 or 250X2) were identified according to one of the definitions below: (1) Diagnostic codes in outpatient visits if the patient had an initial diagnosis at any time during the follow-up period and then had one or more additional diagnoses within the subsequent 12 months. The first and last outpatient visit within 1 year had to be more than 30 days apart to avoid accidental inclusion of miscoded patients; (2) Diagnostic codes had to be in the hospitalization databases at least once during the follow-up period. The index date for each patient of ESRD dialysis group was the date of his or her first time of dialysis in 2000. The index date for patients in the control group was the date they first enrolled in the NHI program. If that date was before 1 January 2000, the index date was set at 1 January 2000.

The age- and gender-specific hazard rates were determined with person-years as the denominator under the Poisson assumption. The cumulative incidence of new-onset diabetic mellitus was calculated. Cox proportional hazards regression models were used to assess the association of ESRD dialysis with the risk of NODM. The independent associations were examined using multivariate analysis with adjusting simultaneously for age, gender, geographic area, urban/rural status and various comorbidities. We adjusted geographic variables for the presence of an urban-rural difference in the accessibility to medical care in Taiwan [13]. The comorbidities considered in our analysis included a number of medical diagnoses considered to pose a risk for DM [4]. All statistical analyses were done using the Statistical Package for Social Sciences 17.0 for Windows (SPSS Inc; Chicago, IL, USA). Significance was set at p < 0.05.

## Results

### Type 2 diabetes status and incident ESRD dialysis

#### Baseline characteristics

There were no significant differences in gender or age between the T2DM and Control groups (Table 1). The number of patients with T2DM increased with increasing age: only 10.3% were 18 and 44 years old, but 41.1% were ≥ 65 years old. Geographical distribution was not significantly different between the two groups, but urbanization levels of residential areas were: 28.5%, 25.7%, and 45.8% of the patients in the diabetes group lived in urban, suburban, and rural areas, respectively; 30.7%, 24.8%, and 44.5% of the controls lived in urban, suburban, and rural areas, respectively. Compared with controls, patients with T2DM had more comorbidities, that were related to ESRD dialysis.

**Table 1:** Demographic data of the type 2 diabetes and control groups.

	Control Group		T2DM Group	
	(n = 82,340)		(n = 20,585)	
	n	(%)	n	(%)
Gender				
Female	42644	(51.8)	10661	(51.8)
Male	39696	(48.2)	9924	(48.2)
Age (years)				
18–44	8520	(10.3)	2130	(10.3)
45–54	16768	(20.4)	4192	(20.4)
55–64	23167	(28.1)	5794	(28.1)
≥ 65	33885	(41.2)	8469	(41.1)
Mean age (SD)	61.1	(12.2)	61.1	(12.2)
Urbanization level of residential area				
Urban	25271	(30.7)	5872	(28.5)
Suburban	20456	(24.8)	5289	(25.7)
Rural	36613	(44.5)	9424	(45.8)
Geographical location of residential area				
North	39866	(48.4)	9119	(44.3)
Center	14785	(18.0)	3775	(18.3)
South	25076	(30.5)	7007	(34.0)
East	2612	(3.2)	684	(3.3)
Risk factors for ESRD dialysis				
Hypertension	15743	(19.1)	10901	(53.0)
Congestive Heart Failure	1262	(1.5)	830	(4.0)
Coronary Artery Disease	4275	(5.2)	2910	(14.1)
Cerebrovascular Disease	2990	(3.6)	2187	(10.6)
Hyperuricemia	1464	(1.8)	1208	(5.9)
Dyslipidemia	1855	(2.3)	2721	(13.2)

ESRD, end-stage renal disease; SD, standard deviation; T2DM, type 2 diabetes.

#### Cox hazard analyses

**Fig 1:**
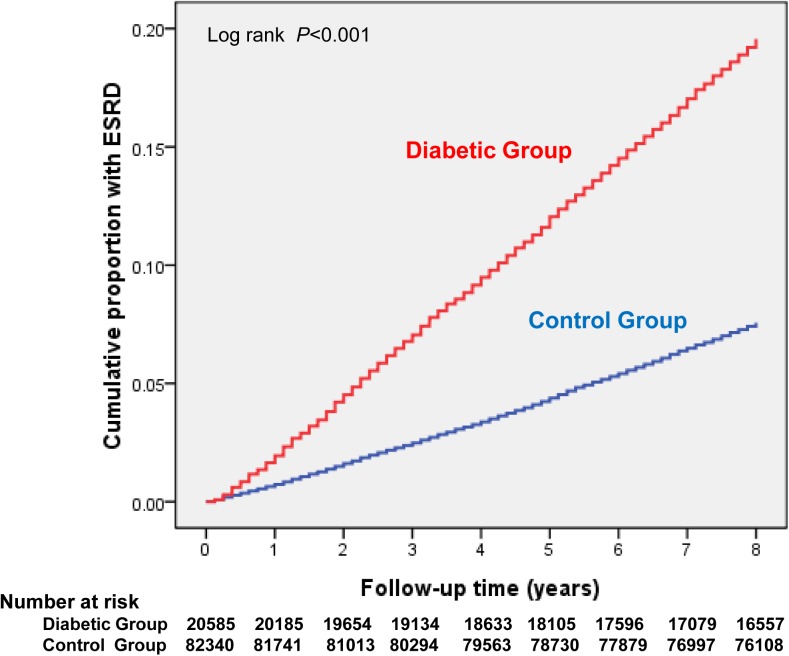
Cumulative incidence of ESRD dialysis by type 2 diabetes cohort and control cohort (general population without diabetes).

During the nearly 9-year study period, there were 419 incident cases of ESRD dialysis among participants without DM and 1093 incident cases among those with T2DM (Fig 1). The crude incidence of ESRD dialysis was 6.78 (95% CI: 6.38–7.19) per 1,000 person-years (PY) for those with T2DM and 0.61 (95% CI: 0.55–0.67) per 1,000 PY for those without DM (Table 2). Patients with T2DM had a much higher incidence of ESRD dialysis than did Controls regardless of age and gender. Moreover, the incidence of ESRD dialysis in Controls tended to increase with age regardless of gender. However, for the patients with T2DM, the age-group with the highest incidence densities (ID) of ESRD dialysis was in the 55- to 64-year-olds (men: 8.51 [95% CI: 7.31–9.86]; women: 7.47 [95% CI: 6.48–8.57]). Compared with control, the change in HR of ESRD dialysis decreased with increasing age regardless of gender. The HR decreased from 20.93 in men 18–44 years old to 5.48 in men ≥ 65 years old, and from 16.54 in women 18–44 years old to 4.84 in women ≥ 65 years old. The significant association between diabetes and incident ESRD dialysis persisted (HR 7.97 [95% CI: 7.05–8.00]) after age, gender, geographical area, urbanization status, and clinical risk factors for ESRD dialysis had been adjusted for.

**Table 2:** Overall and age- and gender-specific relative hazards of end-stage renal disease dialysis in association with type 2 diabetes.

	Control Group			T2DM Group			Adjusted HR (95%CI) in association with diabetes
	Patients	Events	ID (per 1,000 PY)	Patients	Events	ID (per 1,000 PY)	
Variables	(n)	(n)	(95% CI) †	(n)	(n)	(95% CI) †	
Men (years old)							
18–44	4853	10	0.24 (0.12–0.42)	1213	61	6.03 (4.65–7.69)	20.93 (10.47–41.86) §
45–54	8853	26	0.34 (0.23–0.49)	2214	121	6.72 (5.60–8.00)	15.13 (9.63–23.78) §
55–64	10179	48	0.56 (0.42–0.73)	2546	171	8.51 (7.31–9.86)	11.31 (8.03–15.93) §
≥ 65	15811	95	0.75 (0.61–0.91)	3951	165	5.72 (4.90–6.65)	5.48 (4.17–7.19) §
Total	39696	179	0.54 (0.47–0.62)	9924	518	6.72 (6.16–7.32)	9.23 (7.69–11.08) ║
Women (years old)							
18–44	3667	8	0.25 (0.12–0.48)	917	45	5.86 (4.33–7.78)	16.54 (7.52–36.35) §
45–54	7915	26	0.38 (0.25–0.55)	1978	117	7.14 (5.93–8.52)	12.01 (7.61–18.95) §
55–64	12988	67	0.60 (0.47–0.76)	3248	198	7.47 (6.48–8.57)	8.26 (6.13–11.13) §
≥ 65	18074	138	0.92 (0.78–1.09)	4518	215	6.39 (5.57–7.28)	4.89 (3.87–6.18) §
Total	42644	240	0.66 (0.58–0.75)	10661	575	6.83 (6.29–7.40)	7.03 (5.97–8.27) ║
Overall	82340	419	0.61 (0.55–0.67)	20585	1093	6.78 (6.38–7.19)	7.97 (7.05–8.99) ¶

ESRD, end-stage renal disease; T2DM, type 2 diabetes; ID, incidence densities; PY, person-years; CI, Confidence interval; HR, hazard rations.

† Based on Poisson assumption.

§ Based on Cox proportional hazards regression with adjustment of geographic area, urbanization status, and clinical risk factors for ESRD.

║Based on Cox proportional hazards regression with adjustment of geographic area, urbanization status, clinical risk factors for ESRD dialysis, and age.

¶ Based on Cox proportional hazards regression with adjustment of geographic area, urbanization status, clinical risk factors for ESRD dialysis, age and gender

Further interactions were also tested between age/gender and these comorbidities. The complete model, which included all the covariates, was used for Cox regression analysis. Then each interaction term was separately included once at a time. As result, there is a significant interaction: HTN x age (P < 0.01). After stratification, the effect of DM on ESRD dialysis was much more prominent in patients without HTN than those with HTN. Among non-HTN patients, adjusted HR of ESRD dialysis were 18.98 (95% CI: 10.87–33.17), 16.04 (95% CI: 10.97–23.45), 11.72 (95% CI: 8.68–15.81), and 5.83 (95% CI: 4.33–7.85) in 18–44, 45–54, 55–64, and ≥ 65 years old, respectively. Among HTN patients, adjusted HR of ESRD dialysis were 10.08 (95% CI: 2.42–41.99), 9.86 (95% CI: 5.57–17.45), 7.54 (95% CI: 5.44–10.46), and 4.77 (95% CI: 3.84–5.93) in 18–44, 45–54, 55–64, and ≥ 65 years old, respectively.

### ESRD dialysis status and new-onset diabetes

#### Baseline characteristics

There were no differences in gender or age between these 2 groups (Table 3). Geographical distribution was not significantly different between the two groups, but urbanization levels of residential areas were. More than half (77.4%) of the patients in the ESRD dialysis group lived in urban or suburban areas, and almost half of the control (43.5%) lived in urban area. Patients in the ESRD dialysis group were more likely then Controls to have comorbidities, which related to NODM.

**Table 3:** Demographic data of the end-stage renal disease dialysis and control groups.

	Control Group		ESRD Dialysis Group	
	(n = 73,956)		(n = 18,489)	
	n	(%)	n	(%)
Gender				
Female	40092	(54.2)	10023	(54.2)
Male	33864	(45.8)	8466	(45.8)
Age (years)				
18–44	21310	(28.8)	5330	(28.8)
45–54	16356	(22.1)	4086	(22.1)
55–64	15445	(20.9)	3861	(20.9)
≥ 65	20845	(28.2)	5212	(28.2)
Mean age (SD)	54.6	(15.0)	54.6	(15.0)
Urbanization of residential area				
Urban	21731	(29.4)	7069	(38.2)
Suburban	20086	(27.2)	7253	(39.2)
Rural	32139	(43.5)	4167	(22.5)
Geographical location of residential area				
North	36814	(49.8)	8226	(44.5)
Center	13659	(18.5)	3552	(19.2)
South	21212	(28.7)	6299	(34.1)
East	2271	(3.1)	412	(2.2)
Risk factors for diabetes				
Hypertension	10482	(14.2)	8357	(45.2)
Coronary Artery Disease	2636	(3.6)	2112	(11.4)
Cerebrovascular Disease	1876	(2.5)	1087	(5.9)
Dyslipidemia	1148	(1.6)	929	(5.0)

ESRD, end-stage renal disease.

### Cox hazard analyses

**Fig 2:**
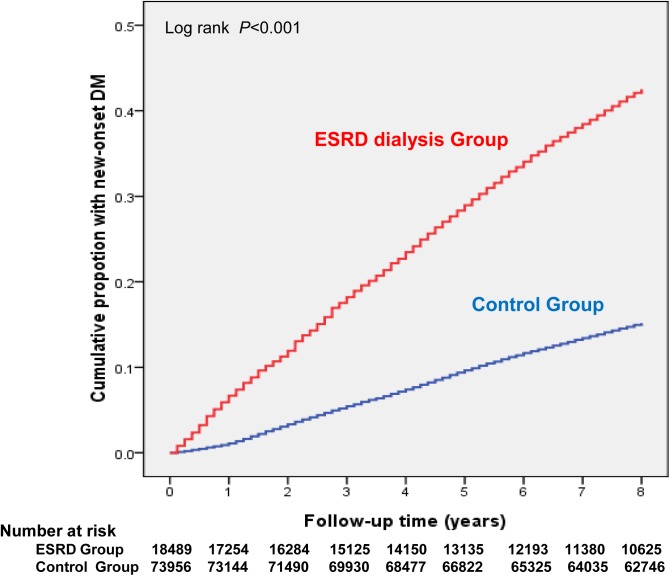
Cumulative incidence of new-onset diabetes by ESRD dialysis cohort and control cohort (general population without ESRD dialysis).

During the nearly 9-year study period, there were 8469 NODM cases in patients not on ESRD dialysis, and 2822 NODM cases in patients on ESRD dialysis (Fig 2). The crude incidence of NODM was 22.84 (95% CI: 22.01–23.70) per 1,000 PY for those on ESRD dialysis, and 13.99 (95% CI: 13.70–14.29) per 1,000 PY for those not on ESRD dialysis (Table 4). Patients on ESRD dialysis had a much higher rate of ID than did Controls regardless of age and gender. Moreover, the highest age-specific ID of NODM for Controls was in the 55- to 64-year-olds (men: 19.24 [95% CI: 18.09–20.45]; women: 21.86 [95% CI: 20.79–22.98]). However, there was a clear tendency of NODM with increasing age in patients on ESRD dialysis. The highest age-specific ID of NODM was in those ≥ 65 years old (men: 31.14 [95% CI: 28.29–34.21]; women: 37.87 [95% CI: 34.95–40.96]). The association between ESRD dialysis and NODM persisted after age, gender, geographical area, urbanization status, and clinical risk factors for NODM had been adjusted for. Patients on ESRD dialysis had a 40% higher risk for NODM (HR: 1.40 [95% CI: 1.34–1.47]).

**Table 4:** Overall and age- and gender-specific relative hazards of new-onset diabetes in association with end-stage renal disease dialysis.

	Control Group			ESRD Dialysis Group			Adjusted HR (95%CI) in association with ESRD dialysis
	Patients	Events	ID (per 1,000 PY)	Patients	Events	ID (per 1,000 PY)	
Variables	(n)	(n)	(95% CI) †	(n)	(n)	(95% CI) †	
Men (years)							
18–44	10687	513	5.52 (5.06–6.01)	2671	187	9.41 (8.13–10.83)	1.17 (0.94–1.46)
45–54	6785	820	14.54 (13.57–15.57)	1697	251	21.39 (18.87–24.17)	1.21 (1.03–1.42) §
55–64	6707	1027	19.24 (18.09–20.45)	1676	298	28.17 (25.11–31.51)	1.24 (1.08–1.43) §
≥ 65	9685	1197	16.18 (15.29–17.12)	2422	426	31.14 (28.29–34.21)	1.62 (1.47–1.79) §
Total	33864	3557	12.86 (12.44–13.28)	8466	1162	20.80 (19.63–22.00)	1.39 (1.29–1.49) ║
Women (years)							
<45	10623	431	4.64 (4.22–5.10)	2659	213	10.67 (9.31–12.18)	1.61(1.30–2.00) §
45–54	9571	1148	14.35 (13.54–15.20)	2389	401	23.68 (21.45–26.09)	1.22 (1.07–1.40) §
55–64	8738	1527	21.86 (20.79–22.98)	2185	435	29.72 (27.02–32.61)	1.16 (1.03–1.30) §
≥ 65	11160	1806	21.01 (20.06–22.00)	2790	611	37.87 (34.95–40.96)	1.62 (1.47–1.79) §
Total	40092	4912	14.95 (14.53–15.37)	10023	1660	24.53 (23.37–25.74)	1.42 (1.33–1.50) ║
Overall	73956	8469	13.99 (13.70–14.29)	18489	2822	22.84 (22.01–23.70)	1.40 (1.34–1.47) ¶

ESRD, end-stage renal disease; ID, incidence densities; PY, person-years; CI, Confidence interval; HR, hazard rations.

† Based on Poisson assumption.

§ Based on Cox proportional hazards regression with adjustment of geographic area, urbanization status, and clinical risk factors for diabetes.

║Based on Cox proportional hazards regression with adjustment of geographic area, urbanization status, clinical risk factors for diabetes, and age.

¶ Based on Cox proportional hazards regression with adjustment of geographic area, urbanization status, clinical risk factors for diabetes, age, and gender.

## Discussion

We used the Taiwan NHIRD, which has health insurance claims data for 99% of the population of Taiwan, to investigate the relationship between DM and ESRD dialysis. This is the first national cohort study to reveal a bidirectional association between DM and ESRD dialysis. Our findings suggest that ESRD dialysis patients have a modestly higher risk of developing NODM during follow-up, independent of age, gender, geographic area, urbanization status, and clinical risk factors for DM. The relationship between patients with T2DM who develop a need for ESRD dialysis was much stronger than between patients with ESRD dialysis who develop NODM.

There is a strong association between T2DM and the risk of developing ESRD dialysis. Kuo et al. reported that patients with diabetes have twice the risk for developing CKD after adjustment for age, gender, regions and comorbidites [14–20]. Pavkov et al [21]. reported that the incidence of diabetic ESRD was 5.4 case per 1000 person-years (95% CI: 4.4–6.4) in patients with diabetes onset between 20–55 years of age. In the present study, the incidence of ESRD dialysis in patients with T2DM was 6.78 per 1000 person-years, which was 10-fold higher than that of Controls.

The prevalence of DM increases with age [22], which is related to insulin resistance and decreased cell function [23]. Our findings are similar. Only 10.3% of our patients with T2DM were 18–44 years old, but 41.1% were older than 65 years. In addition, we found patients with T2DM are likely to have more comorbidities as compared with control group. These comorbidities, including HTN (15), CHF (16), CAD (17), CVA (18), hyperuricemia (19) and dyslipidemia (20), are also risk factors for ESRD dialysis. This disproportion may confound our results. To adjust for potential confounding in the relationship between DM/non-DM individuals and risk factors for ESRD dialysis, multivariate analyses were used to model to ESRD dialysis. After adjustment, patients with T2DM still had nearly eight times at risk of ESRD dialysis than did patients without DM.

The incidence of NODM in ESRD dialysis patients was 22.84 per 1000 person-years in our study. This is similar to what Salifu et al [3]. found in their analysis of the data from the United States Renal Data System: the incidence of NODM after dialysis was 20 per 1000 patient-years. In our study, ESRD dialysis patients were observed to suffer from a much higher incidence than did Controlc, irrespective of age and gender. This finding is consistent with those of a number of previous studies. In the US, the incidence rate for DM is around 0.5–0.9% per year in the general population [7] and 4.6% in the first year after initiation of dialysis [3]. In Taiwan, the incidence rate for DM is around 0.7–0.9% per year in the general population [8] and the cumulative incidence rate of NODM is 4% at one year, 9% at three years, and 14% at five years in dialysis population [4].

The underlying mechanism for the relationship between ESRD dialysis and NODM have not fully understood. The development of DM is associated with β-cell dysfunction and insulin resistance [24, 25]. The characteristics of ESRD dialysis patients, including being older [1] and having multiple cardiovascular diseases [1], are associated with insulin resistance [26, 27]. In our previous analysis using Taiwan`s National Health Insurance Research Database [4], being older and having some comorbidities were independent risk factors for NODM in ESRD dialysis patients [4]. In this study, we found there was a clear tendency for developing NODM with increasing age in patients on ESRD dialysis. The pathogenesis of age-related DM is associated with insulin resistance and decreased cell function [23]. After adjustment for age, gender and comorbidities, ESRD dialysis patients still had a 40% higher risk for NODM than did the general population (HR: 1.40; 95% CI: 1.34–1.47). It is well known that chronic inflammation is common in ESRD dialysis patients [28, 29]. Lin et al. [30] reported that blood sugar is positively associated with inflammatory markers but negatively correlated with nutritional parameters in patients with ESRD on hemodialysis. This finding is supported by Szeto et al. [6], who also reported significant correlations between plasma glucose levels and C-reactive protein level in ESRD patients receiving peritoneal dialysis.

There are several limitations to this study. First, like other studies that have used administrative data, we did not control for some unmeasured confounding variables: measures of adiposity, familial history of DM, nutritional status, biochemical data, body mass index and lifestyle etc., which are important risk factors for DM. Second, because lack of biochemical data, the comorbidities relied only on the claim data and ICD-9-CM diagnosis codes. It is possible that some of the diseases were misclassified. Third, we were unable to take into account the severity of the diseases, which reduced our chances of showing the severity-related effects of comorbidities. Finally, it would be better to describe the data on medications received by subjects; however, there was no information of medications in our database. Further studies needed to be performed to evaluate it.

In conclusion, there is a bidirectional association between ESRD dialysis and T2DM. These observations have an important public health and impact because the global prevalence and incidence of ESRD dialysis and DM have increased markedly during the past few years. However, we did not control for some unmeasured confounding variables, including e.g. measures of adiposity, familial history of diabetes, etc, which are important risk factors for DM. Additional studies to determine the biological mechanism of ESRD dialysis-related NODM are required. These findings of bidirectional association between ESRD dialysis and T2DM suggest that physicians should prevent both conditions and be aware of increased risk of NODM in ESRD dialysis patients.

## Abbreviations

ESRD: end-stage renal disease
NODM: new-onset diabetes mellitus
T2DM: type 2 diabetes
DM: diabetes
NHIRD: National Health Insurance Research Database
